# Environmental Enrichment During Adulthood Reduces Sucrose Binge-Like Intake in a High Drinking in the Dark Phenotype (HD) in C57BL/6J Mice

**DOI:** 10.3389/fnbeh.2019.00027

**Published:** 2019-02-15

**Authors:** Elisa Rodríguez-Ortega, Manuel Alcaraz-Iborra, Leticia de la Fuente, Enedina de Amo, Inmaculada Cubero

**Affiliations:** ^1^Departamento de Psicología, Faculty of Education Sciences, University of Almería, Almería, Spain; ^2^CERNEP, Universidad de Almería, Almería, Spain

**Keywords:** environmental enrichment, drinking in the dark, sucrose, HD/LD phenotypes, orexin receptor 1

## Abstract

Repetitive binge episodes favor transition to binge-eating disorders. Experimental evidence points to positive influence of environmental enrichment (EE) on drug/food addiction, although far less is known regarding EE effects over binge-like consumption. Here, we evaluate the following: (1) whether switching from nonenriched standard environment (SE) to EE housing conditions during adulthood alters a stable pattern of voluntary sucrose (10% w/v) binge-like intake in high (HD) vs. low (LD) drinking phenotypes under a drinking in the dark (DID) schedule; and (2) sucrose binge-like intake in a DID task in response to a pharmacological challenge with an OXr1 antagonist in HD/LD subpopulations after long-term exposure to SE or EE conditions. Adolescent (postnatal day 21; PND21) mice were housed in SE conditions. At PND65, all animals were long-term exposed to sucrose DID. On the first episode of DID (PND65), animals were divided into HD vs. LD subpopulations according to their sucrose intake. On PND85, an OXr1 antagonist test was conducted on HD and LD mice with SB-334867 (SB) administration. On PND95, HD and LD subpopulations were again randomly allocated into two subgroups, resulting in the following experimental conditions: HD-SE, HD-EE, LD-SE and LD-EE. Sucrose binge-like intake continued until PND116, when a second SB test was conducted. The main findings are: (1) a single 2 h episode of sucrose binge drinking in a DID procedure consistently segregates two behavioral subpopulations, HD and LD; (2) when adult mice in standard conditions and long-term exposed to sucrose DID were switched to EE conditions, an immediate reduction in sucrose binge-like intake was observed in HD mice, pointing to a therapeutic role of EE exposure; and (3) administration of the OXr1 antagonist caused an acute reduction in sucrose binge-like intake in HD and LD mice exposed to SE conditions. Importantly, exposure to EE conditions blunted the inhibitory effect of SB on sucrose binge consumption in both behavioral phenotypes, indirectly suggesting a potential EE/OXr1 signaling interaction. We propose the hypothesis that EE might regulate OX-dependent anxiety/compulsivity brain systems, which might secondarily modulate sucrose binge-like intake.

## Introduction

Binge eating disorder (BED) is a severe, life-threatening, addiction spectrum disorder (American Psychiatric Association, 2013) included in the Diagnostic and Statistical Manual of Mental Disorders (DMS-V) that is characterized by recurrent episodes of eating large quantities of food in a discrete period of time (American Psychiatric Association, 2013). BED is the most common eating disorder in the United States, and its study is of major concern given BED comorbidity with obesity (Grucza et al., 2007). Repetitive binge episodes are a common pattern exhibited during early stages in the addiction cycle (Koob and Volkow, 2009), and it has been proposed that repetitive binge eating episodes might favor transition to binge-eating disorders and “food addiction” (Avena, 2010; Cowin et al., 2011; Smith and Robbins, 2013). Therefore, it is important a further knowledge of the neurobehavioral and neurochemical mechanisms involved in repetitive binge eating in order to develop innovative behavioral and pharmacological strategies that may protect binge eaters from transition to eating disorders and food addiction.

Recent growing experimental evidence has showed that environmental enrichment (EE), a preclinical model where animals are exposed to housing conditions with access to exercise, novelty and social interaction (Crofton et al., 2015), which facilitates motor, cognitive and sensory stimulation, may have positive consequences for multiple pathological processes, such as drug addiction (Nithianantharajah and Hannan, 2006; Stairs and Bardo, 2009; Solinas et al., 2010), ethanol consumption, ethanol self-administration (SA) and ethanol reward (Deehan et al., 2007, 2011; de Carvalho et al., 2010; Lopez et al., 2011; Li et al., 2015; Lopez and Laber, 2015; Bahi, 2017; Marianno et al., 2017). Additionally, recent studies suggests that EE might also protect and have therapeutic modulatory effects over the emergence of binge-like consumption. Thus, social and EE reduced ethanol binge-like consumption and preference in C57BL/6 mice in a modified two-bottle choice drinking in the dark task (DID-2BC; Marianno et al., 2017), and 40 days of social housing after weaning caused decreased ethanol binge-like intake in C57BL/6J mice in a DID-2BC schedule when compared with mice in isolated conditions (Lopez et al., 2011). Similarly, EE exposure effectively blunted excessive ethanol binge-like consumption in C57BL/6J mice chronically housed in social isolation conditions (Lopez and Laber, 2015). Our laboratory showed that rearing mice in EE during adolescence effectively reduces high ethanol binge-like intake during adulthood, as measured by an intermittent DID (iDID) procedure, along with reducing anxiety-, compulsivity-like responses and novelty-seeking behaviors, as measured by the hole board (HB) and the elevated plus maze (EPM) tests (Rodríguez-Ortega et al., 2018). Additionally, EE shows therapeutic effects in drug intake by dependent animals (for review, see Solinas et al., 2010). EE also shows protective and therapeutic effects in ethanol binge-like consumption (Lopez et al., 2011; Lopez and Laber, 2015; Marianno et al., 2017; Rodríguez-Ortega et al., 2018).

Recent studies have provided additional evidence that enriched housing conditions modulate consumption of sucrose, a highly palatable substance. Thus, Lister hooded rats reared in social conditions significantly consumed less sucrose in a 2BC schedule than their littermates reared in isolation (Hall et al., 1997); Sprague-Dawley rats housed in isolation conditions significantly drunk more sucrose in 2BC paradigm than their counterparts housed in enriched or social conditions (Brenes and Fornaguera, 2008); sucrose conditioned place preference (CPP) was higher in Wistar rats housed in isolation conditions when compared with rats in social housing conditions (Van den Berg et al., 1999); EE housed Sprague-Dawley rats showed less sensitization to sucrose paired with a cue-light reinforcer that rats housed under social or isolation conditions (Gill and Cain, 2011). Similarly, EE housed Sprague-Dawley rats exhibited enhanced extinction of sucrose-maintained lever presses than rats housed in isolation as measured by a continuous reinforcement paradigm (Stairs et al., 2006). Furthermore, EE exposure reduced sucrose seeking in Long-Evans rats trained for sucrose SA (Grimm et al., 2008); Long-Evans rats chronically or acutely exposure to EE conditions showed blunted sucrose consumption and cue reactivity (Grimm et al., 2013, 2016). Taken together, evidences from recent studies have shown that EE could modulate sucrose seeking and consumption; nevertheless, little is known about EE effects over sucrose binge-like consumption.

Given experimental evidence indicating that EE ameliorates sucrose preference, sucrose intake and sucrose seeking in preclinical models (Stairs et al., 2006; Brenes and Fornaguera, 2008; Grimm et al., 2008, 2013, 2016; Gill and Cain, 2011), we hypothesized that EE might also have a preventive and therapeutic impact on sucrose binge-like consumption as measured by a DID procedure. The first objective in this study addresses the ability of EE to modulate sucrose binge-like intake, as measured by a long-term DID procedure. It is important to highlight that the selected long-term DID procedure fulfills the criteria for preclinical models of binge eating. Thus, the selected DID procedure triggers binge-like consumption of sucrose and saccharin (Lowery et al., 2008, 2010; Kaur et al., 2012) in *ad libitum-fed* animals in such a way that over a period of 2 h, mice drunk on average more than 50% of the total sucrose they usually consume over 24 h, under a 2BC unlimited access schedule (Sparta et al., 2008; Kaur et al., 2012; Alcaraz-Iborra et al., 2014). Preclinical models have been developed based on the DSM-5 criteria for human binge episodes, including eating during a short, discrete period of time larger amounts of food than the normal quantity of food consumed under the same circumstances during the same period of time (Corwin and Babbs, 2012; American Psychiatric Association, 2013). The scientific literature has additionally considered a 2 h binge episode to be an adequate amount of time to assess binging in both human (Wolfe et al., 2009) and rodent studies (Valdivia et al., 2014); during this period, binging animals should consume at least a twofold increase in calories compared with the control group (Perello et al., 2014). Finally, repetitive binge episodes should also be part of animal models of binge eating, since they are a hallmark of BED or BN disorders (American Psychiatric Association, 2013; Perello et al., 2014).

It is worth noting that even though many individuals exposed to addictive substances might exhibit spontaneous binge-like episodes, only a small number of them would develop drug/food addiction and compulsive seeking behaviors after repeated binge-withdrawal-relapse cycles (Belin et al., 2016). Several authors have pointed to the utility of High vs. Low consumer subpopulations in preclinical and clinical studies for a better understanding of the neurobehavioral mechanisms underlying drug addiction and BEDs (for reviews, see Ramoz et al., 2007; Monteleone and Maj, 2008; Hutson et al., 2018). In this regard, we have provided recent behavioral, pharmacological and molecular evidence pointing to the relevance of using the High/Low drinker (HD/LD) subpopulations stemming from DID procedures to explore neurobehavioral processes underlying ethanol binge intake (Alcaraz-Iborra et al., 2017). We defend here the idea that DID might also successfully segregate two behavioral phenotypes (HD/LD) for sucrose binge-like intake. In the study, we comparatively evaluate whether switching from nonenriched standard environment (SE) to enriched environmental housing conditions during adulthood alters a stable pattern of voluntary sucrose binge-like intake as measured by the DID procedure in HD vs. LD subpopulations.

Recent research has underscored the capacity of orexin (OX) in mediating “food addiction,” binge-like eating (Avena et al., 2008; Avena, 2010; Avena and Bocarsly, 2012), compulsive consumption of palatable food (Smith and Robbins, 2013) and binge-like intake of palatable caloric and noncaloric substances (Alcaraz-Iborra et al., 2014). Moreover, it has been suggested the involvement of OXr1 signaling in food-reinforced behaviors (Nair et al., 2008; Borgland et al., 2009; Cason and Aston-Jones, 2013), food seeking (Cason and Aston-Jones, 2013, 2014; Kay et al., 2014) and compulsive eating (see Merlo Pich and Melotto, 2014 ; for review, Piccoli et al., 2012). Our laboratory has provided additional evidence that OXr1 blockade significantly decreases sucrose and saccharin binge-like intake in a DID test in nondeprived animals; furthermore, exposure during 4 days to 2 h episodes of sucrose or saccharin binge-like intake significantly decreases expression of OX mRNA in the lateral hypothalamus (LH; Alcaraz-Iborra et al., 2014), indicating the role of OXr1 signaling in sucrose binge-like consumption. Bearing in mind experimental evidence suggesting a key role for OXr1 signaling in sucrose binge-like consumption in nondependent animals, the third objective in the study was aimed to provide preliminary pharmacological evidence regarding a potential EE/OX system interaction. For this purpose, we evaluated sucrose binge-like intake in a DID task in response to a pharmacological challenge with an OXr1 antagonist in HD/LD mice exposed long-term to EE vs. SE.

## Materials and Methods

### Animals and Housing

Male young C57BL/6J mice (Charles River Laboratories, Spain S.A., Barcelona, Spain) arrived to the laboratory immediately after weaning (PND21) and were housed in in polycarbonate cages (50 × 15 × 25 cm) in groups of four animals (standard housing condition, SE) with stainless steel wire mesh lids and sawdust bedding. To help individual following across the study, each animal’s tail was marked daily with a nontoxic marker. During the entire study, animals had *ad libitum* access to water and rodent chow. Room temperature was set at 21 ± 2°C in a 12:12 h light/dark schedule (lights on from 7 pm to 7 am). All procedures were approved by the Bioethical Animal Care Committee at the University of Almeria, Spain following the animal care guidelines established by the Spanish Royal Decree 53/2013 for reducing animal pain and discomfort.

### Drugs

The OXr1 antagonist SB-334867 (SB; 1-(2-methylbenzoxazol-6-yl)-3-[1,5]naphthyridin-4-yl urea hydrochloridre; Tocris, Bristol, UK) was suspended in 1.5% dimethyl sulfoxide (DMSO), 20% 2-hydroxypropyl-b-cyclodextrin (HBC) and sterile water. For the pharmacological tests, 30 min before the test 5 mg/kg were given intraperitoneally (ip) in a volume of 10 ml/kg (Alcaraz-Iborra et al., 2017). The vehicle solution was prepared with saline and 1.5% DMSO and given in a 10 ml/kg volume. The selected dose of SB effectively reduces ethanol binge-like drinking (Alcaraz-Iborra et al., 2017) and sucrose binge-like drinking (unpublished observation).

### Behavioral Testing

#### Sucrose Binge-Like DID Procedure and Segregation of HD/LD Phenotypes

**Figure 1:**
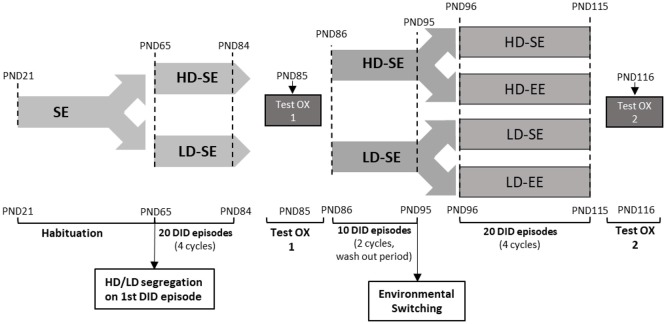
The experimental design over time in the study. After arrival, on PND21, animals were housed in standard environment (SE) until adulthood (PND65). At PND65, mice were exposed to four consecutive drinking in the dark (DID) cycles (from PND65 to PND84). Each DID cycle is composed of five consecutive DID sessions, each one consisting of 2 h access to 10% (w/v) sucrose, 3 h into the dark stage. These DID cycles are repeated consecutively during the whole experiment. Importantly, the first DID session mice segregated High vs. Low DID subpopulations, according to whether their sucrose intake was above or below the median. High/Low drinker (HD/LD) segregation was maintained throughout the rest of the experiment. At PND85, a pharmacological test with SB (5 mg/kg) or vehicle was performed (test OX 1). The drug washout period, from PND86 to PND95, consisted of two additional cycles of DID. At the end of the washout period, on PND95, half of the animals switched their environmental conditions, and half maintained their original housing conditions, leading to four experimental groups: HD-SE, HD-environmental enrichment (EE), LD-SE and LD-EE. From PND96 to PND115, the animal experienced four after-switching additional cycles of sucrose binge-like DID. Finally, on PND116, a new pharmacological challenge with SB (5 mg/kg) or vehicle was performed in all four groups (test OX 2).

On PND65, animals were exposed to 20 consecutive episodes of 10% (w/v) sucrose binge-like DID as follows: 3 h into the dark phase, on days 1–20 animals were transported to individual polycarbonate cages, and a single bottle of 10% (w/v) sucrose was placed on cages for 2 h (see Figure 1). On day 1, HD/LD subpopulations (behavioral phenotypes) were identified in the DID session and segregated by sucrose consumption group median (above median for HD and below median for LD; Alcaraz-Iborra et al., 2017). During sessions, mice had *ad libitum* access to rodent chow, which was daily weighed. To subtract possible fluid spillage from total consumption, an empty cage on each self was used to place dummy bottles to measure lost fluid. Once each DID session ended, mice were returned to their house cages. Every 3 days, body weight (BW) was measured and recorded.

After exposure to 20 episodes of sucrose binge-like DID (PND85), a first pharmacological challenge (Test 1) with an intraperitoneal (ip) administration of the OXr1 antagonist SB (5 mg/kg) or vehicle was performed. Then, all the animals continued a daily sucrose binge-like DID schedule for 10 additional days (drug washout period from PND86 to PND95; see Figure 1).

#### Environmental Enrichment

Once the washout period ended (PND95), HD and LD mice were divided into two subgroups, according to sucrose solution intake, and assigned to one of two possible environmental housing conditions: enriched environment group (EE; *N* = 14) and SE group (*N* = 16). Thus, four experimental groups, EE-HD; EE-LD; SE-HD and SE-LD, emerged, and HD/LD subpopulations were exposed to 20 additional consecutive episodes of sucrose binge-like DID in two different environmental housing conditions, EE vs. SE. Enriched conditions (EE) supposed social housing (four mice per cage) and included a plastic shelter and a mice running wheel permanently available. To maintain novelty, sets of three objects were included every 5 days. Objects consisted in carton tubes and cotton for nesting purposes, checkers tiles, plastic drinking glasses, ping pong balls and PVC tubes. Therefore, EE conditions consisted in access to 5 objects: two of them permanently available and three of them changed every 5 days. On the other hand, the SE maintained previous housing conditions from PND21 to PND95, which consisted of four mice in a cage that contained a single carton tube for nesting purposes.

Finally, on PND116 a second pharmacological challenge (Test 2) with the OX antagonist SB (5 mg/kg, ip) was administered to comparatively evaluate potential EE/OX interaction in HD vs. LD subpopulations (see Figure 1 for details).

### Data Analysis

In the present study, HD/LD subpopulations were selected according to the median of sucrose binge-like intake scores (g/kg/2 h) obtained during the first session of a DID procedure. Thus, mice whose sucrose consumption was above the median constituted the HD subpopulation, and mice whose sucrose consumption was below the median constituted the LD subpopulation (Alcaraz-Iborra et al., 2017). A one-way analyses of variance (ANOVA) evaluated subpopulation differences in sucrose consumption. Data for the average sucrose binge-like intake (g/kg/2 h) and average kilocalories ingested (kcal/g/2 h; chow + sucrose solution) that was obtained both before and after providing EE DID sessions to HD/LD animals were collapsed into 5-day cycles, except for the 10 days of the washout period that were not collapsed in order to evaluate SB washout daily progression. HD and LD sucrose binge-like intakes obtained during the first four cycles of DID before the pharmacological challenge were analyzed as data from before providing EE. Average sucrose binge-like intake and average kilocalories consumed during the additional 5-day cycles of DID after providing EE housing conditions on PND95 were also analyzed to evaluate the differential impact of EE introduction on sucrose binge-like DID consumption by HD and LD subpopulations. Subpopulation differences in average sucrose binge-like intake and kilocalorie consumption during DID sessions while housed in SE conditions prior to environmental switching were analyzed by independent repeated measures (2 × 4; subpopulation × cycle) ANOVAs (from PND65 to PND84; see Figure 1). Independent repeated measures (2 × 10; subpopulation × time) ANOVAs were conducted for sucrose binge-like intake during the washout period (from PND86 to PND95) in order to track consumption by HD/LD animals during that period. To compare sucrose/kcal intake before vs. after EE access, a time point represented by average sucrose/kcal consumed during the last day of the washout period (PND95) was included in the analysis as a before EE estimation. Thus, to evaluate the impact of switching to an enriched environment on sucrose binge-like intake and kilocalorie consumption by HD/LD subpopulations (from PND96 to PND115; see Figure 1), independent repeated measures (2 × 2 × 5; environment × subpopulation × cycle) ANOVAs were performed. BW (g) was also recorded every 3 days, and independent repeated measures ANOVAs were performed for data from before EE (2 × 4; subpopulation × cycle) and after environmental switching (2 × 2 × 4; environment × subpopulation × cycle). All data collected in this study are presented as the mean ± SEM, and differences between and within groups were analyzed using ANOVA procedures. We assessed the assumptions of the ANOVA models on each set of data. The Shapiro-Wilk test of normality and Levene’s test of homoscedasticity were used in all cases; Mauchly’s test of sphericity was used when repeated measures variables were involved in the ANOVA procedure. Thus, when assumptions were violated, the Greenhouse-Geisser correction over the degrees of freedom was used to estimate both the repeated measures and interaction effects. For these results, we only report the violations of the assumptions, indicating the alternative test used when needed. Statistically significant interaction effects were analyzed by the simple effects analysis, and pairwise comparisons of means were performed using the Bonferroni correction. Additionally, in order to evaluate temporal dynamics of consumption over time, planned *t*-tests were performed across key cycles within each group, which allowed a general sight of sucrose consumption progression across the cycles in each group. Thus, we compared cycle 1 vs. 2 and cycle 2 vs. 4 for before EE conditions. We also compared the last washout time-point (PND95) vs. cycle 1, the washout time-point (PND95) vs. cycle 4, and cycle 2 vs. 4 during the conditions after environmental switching. Different error terms were used for each set of comparisons. Additional planned *t*-test comparisons compared binge-like sucrose consumption by vehicle- and SB-injected mice in HD/LD groups in Test 1 and Test 2. In all cases, *p* < 0.05 (two-tailed) was used as the level of statistical significance. Effect size estimates by *η*^2^ (eta-squared) are reported for the significant effects; the effect size indicators provide an estimate of the magnitude of the relationship between the IV and DV that is independent of the sample size. All analyses were made using IBM SPSS (v.22).

## Results

### Sucrose Binge-Like Intake by HD and LD Phenotypes in a Continued DID Schedule

**Figure 2:**
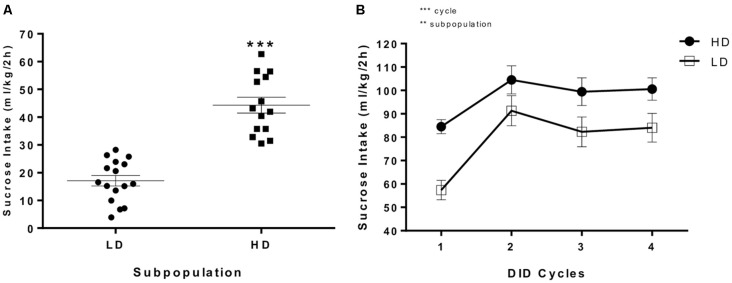
**(A)** Individual 10% (w/v) sucrose binge-like intake (ml/kg/2 h) during the first DID session at PND65 in which HD and LD subpopulations were segregated and **(B)** average (means ± SEM) 10% (w/v) sucrose binge-like intake (ml/kg/2 h) during the first four DID cycles before providing EE (from PND65 to PND84) by HD and LD mice under SE housing conditions. **p < 0.01, ***p < 0.001.

Figure 2A depicts individual sucrose intake data (g/kg/2 h) in the first DID session relative to the median value. The DID procedure clearly segregated the population into two subpopulations for sucrose binge-like consumption, high vs. low drinkers; the one-way ANOVA conducted showed that HD mice consumed significantly more sucrose than their LD counterparts (*F*_(1,30)_ = 68.845, *p* < 0.0001).

Figure 2B shows sucrose binge-like intake by HD and LD subpopulations during the first four 5-day cycles, prior to providing EE, as measured in a DID procedure in SE housing condition (from PND65 to PND84). The assumptions analysis for mixed 2 × 4 ANOVA (subpopulation × cycle) showed that the assumption of sphericity, assessed by Mauchly’s test of sphericity, had been violated ($\chi_{\left(5\right)}^{2}$ = 0.449, *p* = 0.001). The results indicated statistically significant differences for the “cycle” main factor (*F*_(2.30,64.59)_ = 30.61, *p* < 0.0001; *η*^2^ = 0.52) and the “subpopulation” main factor (*F*_(1,28)_ = 7.15, *p* = 0.01; *η*^2^ = 0.20), indicating a stable lower sucrose consumption in LD vs. HD subpopulation over time.

An additional set of planned *t*-test comparisons permitted us to analyze the temporal consumption dynamic over cycles in each experimental group. Thus, the HD group showed statistically significant differences in sucrose consumption in cycle 1 vs. 2 (*t* = −5.07, *p* < 0.001; *df* = 13) but not for cycle 2 vs. 4 (*t* = 0.69, *p* = 0.5; *df* = 13), indicating that sucrose binge-like consumption in HD mice increased from cycle 1–2, reaching a plateau at this time point. For the LD group, planned *t-tests* showed statistically significant differences for cycle 1 vs. 2 (*t* = −7.52, *p* < 0.001; *df* = 15) but not for cycle 2 vs. 4 (*t* = 1.73, *p* = 0.1; *df* = 15), indicating that sucrose binge-like intake by LD mice also increased from cycle 1–2 and then stabilized.

For the kilocalorie intake data, Mauchly’s test for the repeated measures ANOVA 2 × 4 (subpopulation × cycle) showed violation of the sphericity assumption (Mauchly’s test: $\chi_{\left(5\right)}^{2}$ = 0.626, *p* = 0.02). When animals were housed in SE conditions during four sucrose DID cycles (from PND65 to PND86), the ANOVA conducted on kilocalorie data consumed during the DID tests revealed that only the main factor “cycle” attained statistically significant differences (*F*_(2.28,63.88)_ = 9.21, *p* < 0.0001; *η*^2^ = 0.24), indicating increased kilocalorie intake over time in both HD and LD mice (Table 1).

**Table 1:** Average (means ± SEM) kilocalorie intake (g/kg) before and after providing environmental enrichment (EE) housing conditions in both High/Low drinker (HD and LD) subpopulations.

	DID cycles before providing EE			
Group	1	2	3	4
HD	1.91 ± 0.1	1.85 ± 0.12	2.11 ± 0.14	2.26 ± 0.09
LD	1.75 ± 0.1	1.67 ± 0.07	1.89 ± 0.1	1.99 ± 0.11
	**DID cycles after providing EE**			
	**1**	**2**	**3**	**4**
HD-SE	2.17 ± 0.15	2.03 ± 0.19	2.45 ± 0.23	2.25 ± 0.23
HD-EE	2.02 ± 0.16	1.45 ± 0.12	1.79 ± 0.12	1.55 ± 0.15
LD-SE	1.98 ± 0.23	1.77 ± 0.21	1.99 ± 0.19	1.75 ± 0.22
LD-EE	1.83 ± 0.21	1.42 ± 0.25	1.7 ± 0.27	1.55 ± 0.32

*Top panel: average (means ± SEM) kilocalorie intake (g/kg) of HD and LD groups during the four drinking in the dark (DID) cycles before providing EE. Bottom panel: average (means ± SEM) kilocalorie intake of HD-standard environment (SE), HD-EE, LD-SE and LD-EE groups during the four DID cycles after providing EE*.

When BW was analyzed for the first four cycles, Mauchly’s test for the repeated measures ANOVA 2 × 4 (subpopulation × cycle) showed violation of the sphericity assumption (Mauchly’s test: $\chi_{\left(5\right)}^{2}$ = 0.122, *p* < 0.0001). The results showed statistically significant differences for the main factor “cycle” (*F*_(1.46,41.06)_ = 33.44, *p* < 0.0001; *η*^2^ = 0.54) and for the “cycle” × “subpopulation” interaction (*F*_(1.46,41.06)_ = 4.28, *p* = 0.03; *η*^2^ = 0.13). Additional Bonferroni *post hoc* analyses for the interaction showed a progressive increase in BW in HD mice over time (cycle 1 vs. 2, *p* = 0.01; cycle 1 vs. 3, *p* < 0.0001; cycle 1 vs. 4, *p* < 0.0001). For BW in LD mice, the Bonferroni test showed differences in BW in cycle 1 vs. 2 (*p* < 0.0001) and 1 vs. 3 (*p* < 0.0001) but not 1 vs. 4, indicating that BW progressively increased in the HD but not the LD group over time (Table 2).

**Table 2:** Average (means ± SEM) body weight (g) before and after providing EE housing conditions in both HD and LD mice.

	DID cycles before providing EE			
Group	1	2	3	4
HD	26.32 ± 0.48	26.71 ± 0.48	27.37 ± 0.49	27.84 ± 0.5
LD	26.95 ± 0.41	27.29 ± 0.42	27.68 ± 0.42	27.68 ± 0.34
	**DID cycles after providing EE**			
	**1**	**2**	**3**	**4**
HD-SE	29.24 ± 0.89	29.57 ± 0.95	29.84 ± 1.02	29.77 ± 0.81
HD-EE	29.26 ± 0.47	29.31 ± 0.34	29.28 ± 0.69	30.03 ± 0.49
LD-SE	28.91 ± 0.72	29.42 ± 0.72	29.38 ± 0.84	30.36 ± 0.8
LD-EE	28.93 ± 0.52	29.3 ± 0.47	29.49 ± 0.48	29.93 ± 0.48

*Top panel: average (means ± SEM) body weight (g) of HD and LD groups during four DID cycles before providing EE. Bottom panel: average (means ± SEM) body weight (g) of HD-SE, HD-EE, LD-SE and LD-EE groups during four DID cycles after providing EE*.

### Effects of Switching to an Enriched Environment on Sucrose Binge-Like Intake by HD and LD Phenotypes

**Figure 3:**
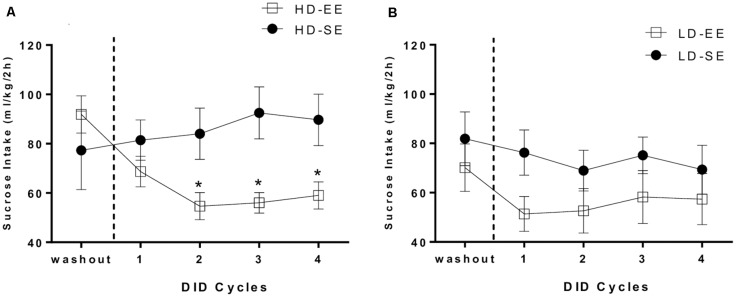
**(A)** HD group average (means ± SEM) 10% (w/v) sucrose binge-like intake (ml/kg/2 h) during the last day of the washout period (PND95) and four DID cycles after providing EE (from PND96 to PND115) and **(B)** LD group average (means ± SEM) 10% (w/v) sucrose binge-like intake (ml/kg/2 h) during the last day of the washout period (PND95) and four DID cycles after providing EE (from PND96 to PND115). Vertical dotted lines indicate the switching time point of the environmental housing conditions. *P < 0.05 relative to HD-SE group.

Figures 3A,B depicts HD and LD sucrose binge-like intake by SE and EE groups during four 5-day DID cycles, from PND96 to PND115. Average sucrose binge-like intake during the last day of the washout period following SB administration (PND95) was included in the analysis as a before EE time-point to analyze the immediate impact of switching to EE on sucrose consumption.

The assumptions analysis for the mixed 2 × 2 × 5 ANOVA (subpopulation × environment × cycle) showed violation of the sphericity assumption (Mauchly’s test: $\chi_{\left(9\right)}^{2}$ = 0.043; *p* = 0.000). The results showed statistically significant differences for the “cycle” (*F*_(1.52,39.67)_ = 4.96, *p* = 0.01; *η*^2^ = 0.16) and “environment” (*F*_(1,26)_ = 4.64, *p* = 0.04; *η*^2^ = 0.15) main factors, as well as for their interaction (environment × cycle; *F*_(1.52,39.67)_ = 4.71, *p* = 0.02; *η*^2^ = 0.15). The second order interaction factor (subpopulation × environment × cycle) also attained statistical significance (*F*_(1.52,39.67)_ = 4.13, *p* = 0.03; *η*^2^ = 0.13). Thus, sucrose binge-like intake for each subpopulation, HD and LD, was analyzed separately. For HD mice, an independent repeated measures ANOVA (2 × 5; environment × cycle) revealed statistically significant differences for factors interaction (*F*_(1.45,17.46)_ = 6.34, *p* = 0.01; *η*^2^ = 0.34; Figure 3A); additional *post hoc* analyses revealed higher sucrose binge-like intake in HD-SE mice compared to HD-EE mice in cycle 2 (*p* = 0.02), cycle 3 (*p* = 0.007) and cycle 4 (*p* = 0.02). Additional planned *t*-test comparisons within HD-SE and HD-EE groups allowed us to evaluate consumption dynamics of each group. Thus, the HD-SE group showed no statistically significant differences in PND95 (before EE time-point) vs. cycle 1 (*t* = −0.343, *p* = 0.74; *df* = 6), in PND95 vs. cycle 2 (*t* = −4.42, *p* = 0.67; *df* = 6), or in cycle 2 vs. 4 (*t* = −2.18, *p* = 0.07; *df* = 6), showing that the HD-SE group had similar sucrose binge-like intake during the periods of the experiment before and after switching. When the HD-EE group was analyzed, planned *t*-test comparisons showed statistically significant differences for PND95 vs. cycle 1 (*t* = 6.05, *p* = 0.001; *df* = 6) and PND95 vs. cycle 2 (*t* = 5.44, *p* = 0.002; *df* = 6), but not for cycle 1 vs. 2 (*t* = 1.91, *p* = 0.1; *df* = 6) or cycle 2 vs. 4 (*t* = −0.81, *p* = 0.44; *df* = 6), showing that EE access immediately and effectively reduced sucrose binge-like intake in cycle 1 in HD mice. Furthermore, reduced sucrose binge-like intake was maintained for the rest of the experiment.

On the other hand, the analysis performed on sucrose binge-like intake data in LD mice did not indicate statistically significant effects for the “cycle” (*F*_(1.57,22.06)_ = 3.56, *p* = 0.06; *η*^2^ = 0.2) or “environment” (*F*_(1,14)_ = 1.77, *p* = 0.2; *η*^2^ = 0.11) factors or for their interaction, indicating that introducing EE housing conditions to LD subpopulation did not significantly alter sucrose binge-like intake (Figure 3B).

For kilocalorie intake, Mauchly’s test for the repeated measures ANOVA (2 × 2 × 5; subpopulation × environment × cycle) showed violation of the sphericity assumption (Mauchly’s test: $\chi_{\left(9\right)}^{2}$ = 0.068, *p* < 0.0001). The analysis conducted on kilocalorie intake data after environmental switching in HD and LD mice showed statistical significance for the main factor “cycle” (*F*_(1.82,47.34)_ = 8.17, *p* = 0.001; *η*^2^ = 0.23) and the interaction “cycle” × “environment” (*F*_(1.82,47.34)_ = 3.94, *p* = 0.02; *η*^2^ = 0.13). Additional Bonferroni *post hoc* analyses revealed higher kilocalorie intake by SE animals than by EE mice in cycle 3 (*p* = 0.03; Table 1).

For BW data analysis, Mauchly’s test for the repeated measures ANOVA (2 × 2 × 5; subpopulation × environment × cycle) showed violation of the sphericity assumption (Mauchly’s test: $\chi_{\left(5\right)}^{2}$ = 0.438, *p* = 0.001). The analysis conducted on BW data from after environmental switching only showed a statistically significant difference for the “cycle” main factor (*F*_(2.15,56.14)_ = 20.96, *p* < 0.0001; *η*^2^ = 0.44), indicating that BW increased over time, regardless of the subpopulation or housing conditions (Table 2).

### Effects of SB-334867 (5 mg/kg) on Sucrose Binge-Like Intake by HD and LD Phenotypes in SE vs. EE Conditions

#### SB Challenge Before Introduction of EE Conditions (PND85; Test OX 1)

**Figure 4:**
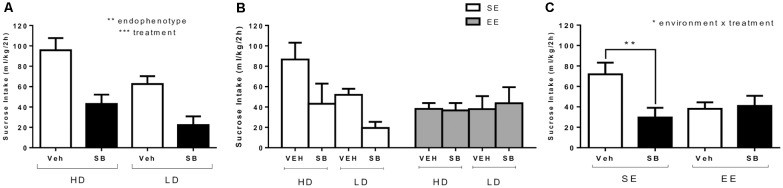
**(A)** Average (means ± SEM) 10% (w/v) sucrose binge-like intake (ml/kg/2 h) by HD and LD mice in response to a pharmacological challenge with SB (5 mg/kg) or vehicle (test 1, PND85) performed after the first four cycles of sucrose DID; **(B)** average (means ± SEM) 10% (w/v) sucrose binge-like intake (ml/kg/2 h) by HD and LD subpopulations in SE/EE conditions in response to a pharmacological challenge with SB (5 mg/kg) or vehicle (test 2, PND116); and **(C)** average (means ± SEM) 10% (w/v) sucrose binge-like intake (ml/kg/2 h) by SE and EE mice in response to a pharmacological challenge with SB (5 mg/kg) or vehicle (test 2, PND116), right after complete four after-switching cycles of sucrose binge-like DID. *p < 0.05, **p < 0.01, ***p < 0.001.

Figure 4A shows average sucrose binge-like intake by HD and LD subpopulations on PND85 (SE, before providing EE housing conditions) as a result of a pharmacological challenge with ip SB (5 mg/kg) or vehicle administration (test OX 1, see Figure 1). An independent (2 × 2; subpopulation × treatment) ANOVA was conducted on sucrose binge-like intake data in response to administration of SB or vehicle by HD and LD subpopulations pre-exposed to four cycles of sucrose DID. The results showed statistically significant differences for “treatment” (*F*_(1,26)_ = 24.88; *p* = 0.000; *η*^2^ = 0.49) and “subpopulation” main factors (*F*_(1,26)_ = 8.32; *p* = 0.008; *η*^2^ = 0.24) but not for their interaction (*F*_(1,26)_ = 0.44, *p* = 0.51; Figure 4A), indicating that SB (5 mg/kg) administration significantly reduced sucrose binge-like intake both in HD and LD subpopulations.

To control the temporal dynamics of sucrose binge consumption during the washout period following OXr1 antagonist administration, daily sucrose binge-like intake during the 10 days of the washout period was analyzed in a repeated measures ANOVA 2 × 10 (subpopulation × time; Table 3) and showed violation of the sphericity assumption (Mauchly’s test: $\chi_{\left(44\right)}^{2}$ = 0.002, *p* < 0.0001). The ANOVA conducted on sucrose binge-like intake data by HD and LD subpopulations revealed statistically significant differences for the main factor “time” (*F*_(3.88,93.34)_ = 2.97, *p* = 0.02; *η*^2^ = 0.11) and “subpopulation” (*F*_(1,24)_ = 7.45, *p* = 0.01; *η*^2^ = 0.23), indicating that differences in sucrose consumption by HD vs. LD persisted over the washout period.

**Table 3:** Average (means ± SEM) sucrose binge-like intake of HD and LD groups during the 10 days of washout period after test OX 1 with SB-334867 administration.

		Washout day				
Group	Treatment	1	2	3	4	5
HD	Veh	112.12 ± 6.26	95.82 ± 7.82	94.22 ± 5.34	85.17 ± 6.09	79.02 ± 9.53
	SB	99.34 ± 5.29	90.83 ± 8.93	99.57 ± 8.92	87.33 ± 10.01	93.56 ± 9.79
LD	Veh	75.67 ± 8.82	68.83 ± 8.19	63.05 ± 6.85	54.65 ± 9.31	63.69 ± 10.95
	SB	67.01 ± 11.18	76.34 ± 12.44	87.79 ± 10.52	78.67 ± 14.74	70.93 ± 14
		**6**	**7**	**8**	**9**	**10**
HD	Veh	81.8 ± 7.66	82.68 ± 5.21	69.99 ± 14.77	82.6 ± 8.98	88.37 ± 6.89
	SB	87.2 ± 12.29	90.38 ± 10.48	76.69 ± 15.07	84.46 ± 12.87	80.78 ± 16.68
LD	Veh	52.86 ± 10.81	51.16 ± 8.1	58.34 ± 9.55	59.63 ± 6.82	62.79 ± 7.09
	SB	67.78 ± 13.76	75.26 ± 11.5	73.25 ± 11.09	92.48 ± 12.03	90.26 ± 12.53

#### SB Challenge After Providing EE Conditions (PND116): Test OX 2

Figure 4B depicts after-switching sucrose binge-like intake by HD/LD subpopulations in SE vs. EE housing conditions as a result of SB (5 mg/kg) and vehicle administration on PND116 (test OX 2, see Figure 1). The assumptions analysis carried out for the independent three-way (2 × 2 × 2; subpopulation × environment × treatment) ANOVA on sucrose binge-like intake in OX test 2 showed compliance with both normality and homoscedasticity assumptions for all of the experimental design conditions. The (2 × 2 × 2) ANOVA performed on sucrose binge-like intake data showed statistically significant differences only for the interaction “environment” × “treatment” (*F*_(1,22)_ = 4.61; *p* = 0.04; *η*^2^ = 0.17), indicating similar sucrose consumption patterns in response to SB by HD and LD mice but differences in response to treatment, depending upon the environmental housing conditions. Thus, since the “subpopulation” factor did not reach statistical significance, HD and LD data were collapsed (Figure 4C). Additional Bonferroni *post hoc* analysis revealed that compared to vehicle, SB reduced sucrose binge-like intake in SE (*p* = 0.01) but not EE mice (*p* = 0.87) in both HD and LD subpopulations (Figure 4C). Additional planned *t*-test comparisons showed higher sucrose intake by SE vs. EE vehicle-injected mice (*t* = 2.65, *p* = 0.02; *df* = 13) but not SB-injected mice (*t* = −0.82, *p* = 0.42; *df* = 13), confirming that EE mice consumed significantly less sucrose than their SE counterparts.

## Discussion

The relevant findings in the current study are the following: (1) a single 2 h episode of sucrose binge drinking in a DID procedure segregates two behavioral subpopulations, HD and LD, in iC57BL/6J mice; (2) when adult mice reared in SE conditions and long-term exposed to sucrose DID (4, 5-day cycles) were switched to EE conditions, an immediate significant reduction in sucrose binge-like intake was observed in HD mice, suggesting the therapeutic role of EE exposure for HD mice showing spontaneous high sucrose binge-like intake; and (3) administration of the OXr1 antagonist SB caused an acute reduction in sucrose binge-like intake in HD and LD mice exposed to SE conditions. Importantly, exposure to EE conditions blunted the inhibitory effect of SB on sucrose binge consumption in both subpopulations, indirectly suggesting that EE exposure might impact the OX system.

Importantly, the therapeutic effects of EE over sucrose consumption from our study were found when compared with control SE housed mice. Interestingly, previous studies have showed that isolated rats (as the EE control condition) had greater sucrose consumption (Hall et al., 1997; Brenes and Fornaguera, 2008). Altogether, current and previous studies results pointing to an isolation and SE conditions correlation with sucrose intake, suggest that social housing (as in our SE group) is a necessary but not sufficient factor to blunt sucrose binge-like intake in mice. However, different methodological variables such as differences in EE housing items or conditions, time to EE and sucrose consumption exposure, together with other factors such as species, sex or strains used for the studies could all influence behavioral results in studies employing EE paradigms (Crofton et al., 2015). Accordingly, disparity between laboratories outcomes as to the EE modulation of sucrose intake might be explained.

Due to both the utility of subpopulations in preclinical and clinical studies for a better understanding of vulnerability to development of drug addiction and BEDs (for reviews, see Ramoz et al., 2007; Monteleone and Maj, 2008; Hutson et al., 2018) as well as our recent behavioral, pharmacological and molecular evidence pointing to the existence of High/Low ethanol drinking (HD/LD) subpopulations stemming from DID procedures (Alcaraz-Iborra et al., 2017), the first objective in the present study addressed the ability of DID to segregate two independent behavioral HD/LD subpopulations for sucrose binge-like consumption, as it does for ethanol binge-like consumption. The first relevant finding is that a single 2 h episode of DID sucrose effectively segregated two behavioral phenotypes, HD and LD that last for four cycles and the entire washout period. Thus, under SE conditions, HD mice showed significantly higher sucrose binge-like intake compared to their LD counterparts before switching to EE. Taken into account that C57BL/6J mice share same genetic load, it is reasonable that HD/LD subpopulations could result from an epigenetic mechanism. Consistently, previous studies have showed elevated variation of phenotypes for ethanol binge-like intake (Crabbe et al., 2010; Barkley-Levenson and Crabbe, 2014) and obesity induced by diet in C57BL/6J mice (Koza et al., 2006). Present results extend our previous observations that DID segregates two behavioral HD/LD phenotypes for spontaneous ethanol binge-like drinking. Moreover, we provide here experimental evidence that HD/LD segregation successfully predicts sucrose consumption in response to environmental variations, as we discuss later. In our laboratory we are currently conducting studies aiming to further characterize HD/LD subpopulations from a molecular and behavioral approach to extend our knowledge on neurobehavioral phenotypes underlying spontaneous high binge intake of caloric palatable food.

The second main consideration in the study is the therapeutic effect of EE access during adulthood on sucrose binge-like intake in HD animals. When adult mice housed during their adolescence in SE conditions and long-term exposed to repetitive sucrose binge episodes in a DID task (4, 5-day cycles) were exposed to EE, an instantaneous decrease in sucrose binge-like intake by HD mice was observed. Thus, HD animals exposed to EE conditions (HD-EE) showed a dramatic and immediate reduction in sucrose binge-like intake, as early as cycle 2, when compared to animals in SE conditions (HD-SE). Moreover, sucrose binge-like consumption by HD-EE animals was reduced immediately and long-term, matching that exhibited by LD (both LD-EE and LD-SE) mice, which confirms and extends previous observations suggesting a therapeutic effect of EE on sucrose consumption and sucrose seeking. EE access had a dramatic effect on both extinction and the response to a sucrose SA-associated cue in rats (Grimm et al., 2008); brief exposure to EE reduced sucrose cue-reactivity and consumption in rats after 1 or 30 days of compelled abstinence from SA (Grimm et al., 2013), and either acute or chronic EE access decreased c-Fos levels in relapse-related rat brain regions (Grimm et al., 2016). Additionally, current results are in concordance with previous preclinical studies supporting therapeutic effects of EE housing conditions on addiction to several drugs such as cocaine (Solinas et al., 2008; Chauvet et al., 2009, 2011, 2012; Thiel et al., 2009), heroin (Galaj et al., 2016), methamphetamine (Hajheidari et al., 2015, 2017) and ethanol (Lopez et al., 2011; Lopez and Laber, 2015; Marianno et al., 2017).

More importantly, our present observations extend and complement recent results from our laboratory showing that EE access during adulthood reduces ethanol binge-like consumption by animals housed in SE conditions during adolescence (Rodríguez-Ortega et al., 2018). Taking together, our present and previous results demonstrate that EE exposure successfully modulates high binge-like intake of a rewarding stimulus, such as ethanol and sucrose, characteristic of the early and transient stages of the addiction cycle (Koob and Volkow, 2009). Additionally, present findings highlight the relevance of segregating behavioral HD/LD subpopulations stemming from DID procedures to evaluate and characterize the therapeutic effects of EE in preclinical models of binge-like intake.

Total kilocalories (sucrose + chow) during DID sessions along with BW were assessed in the study. Despite HD-SE mice consuming more sucrose and total kilocalorie during the study, however, no significant differences were found in BW compared to HD-EE mice or LD mice. Present BW data are in agreement with previous evidence in preclinical studies showing that sweet or fat bingeing procedures fail to induce overweight (Corwin and Babbs, 2012), probably due to a compensatory reduction of chow intake between binge episodes (Avena, 2007; Cowin et al., 2011; Hone-Blanchet and Fecteau, 2014). Furthermore, BW data are consistent with human research showing that only 35% of binge eaters are overweight or obese (Cowin et al., 2011) as well as human studies indicating the existence of a compensatory mechanism triggered in BED in which BW is successfully balanced by undereating after binge episodes (American Psychiatric Association, 2013). In our study, we cannot rule out that longer exposure to sucrose consumption might eventually trigger increased BW in HD-SE mice. Additional studies extending the number of sucrose DID episodes would clarify whether BW consistently varies in parallel with sucrose and caloric consumption under different housing conditions.

The third relevant result is focused on the potential EE/OX interaction. When HD and LD mice housed in SE conditions and pre-exposed long-term to sucrose binge intake were pharmacologically challenged with the OXr1 antagonist SB (test OX 1), sucrose binge-like intake was reduced in both subpopulations to approximately one-half of that exhibited by SE vehicle-injected mice. Interestingly, switching to EE blunted the inhibitory effect of SB, both in HD and LD subpopulations. Although spontaneous sucrose binge intake was low in EE exposed mice at the time of SB second challenge (OX test 2), a floor effect is unlikely since SB successfully reduced sucrose consumption during the OX test 1 in LD mice showing similar levels of intake to that exhibited by EE mice during the OX test 2. Present findings showing an inhibitory modulation of sucrose binge intake by antagonizing OXr1 are in agreement with previous studies supporting a key role for the OX system in food-reward behaviors (Cason and Aston-Jones, 2013, 2014), binge intake (Alcaraz-Iborra et al., 2014) and food overconsumption (Piccoli et al., 2012; Merlo Pich and Melotto, 2014). It is difficult to form any consistent conclusions about the lack of SB effect during the OX test 2. Our observation that EE exposure blunts the inhibitory effect of the OXr1 antagonist SB on sucrose binge intake during a DID test indirectly suggests a role for OXr1 signaling in EE effects. Given the role of OX in anxiety/compulsivity (Piccoli et al., 2012; Merlo Pich and Melotto, 2014) and binge-like consumption (Alcaraz-Iborra et al., 2014), as well as evidence that EE modulates anxiety-like responses (Benaroya-Milshtein et al., 2004; Peña et al., 2006, 2009; Sztainberg et al., 2010; Ragu Varman and Rajan, 2015; Bahi, 2017; Rodríguez-Ortega et al., 2018) and compulsivity (Bechard and Lewis, 2016; Bechard et al., 2016; Rodríguez-Ortega et al., 2018), a working hypothesis is that EE interacts with the OX brain system to primarily modulate anxiety/compulsivity responses; in turn, these responses secondarily reduce binge-like consumption during early, pre-dependent stages of the addiction cycle. Nonetheless, whether present findings indicate the existence of a causal link between EE-driven altered responses to OXr1 antagonism and reduced sucrose consumption by HD mice remains unknown and needs further exploration.

In summary, first, our findings highlight the importance of HD/LD segregation resulting from DID procedures to further evaluate neurobehavioral processes governing the early and transient phases of the food addiction cycle and BED development in preclinical models. Current results underpin the therapeutic value of EE on sucrose binge-like intake in HD mice in a long-term DID procedure that successfully models human binge-like consumption. When HD mice had access to EE housing conditions, sucrose binge-like intake was reduced immediately and in the long term, matching the level of intake exhibited by LD mice. Interestingly, EE blunted the inhibitory effect of an OXr1 antagonist on sucrose consumption, providing preliminary indirect evidence of EE/OX interaction. We suggest the preliminary hypothesis that a primary interaction of EE is on OX-dependent compulsivity/anxiety brain systems, which, secondarily, influence sucrose binge-like consumption during the pre-dependent phases of the addiction cycle.

## Author Contributions

IC provided the overall coordination and supervision for the study. IC and ER-O were responsible for the study concept and design and wrote the manuscript. ER-O, EA and MA-I conducted behavioral characterization. LF was responsible for statistical analyses. All authors critically reviewed the content and approved the final version for publication.

## Conflict of Interest Statement

The authors declare that the research was conducted in the absence of any commercial or financial relationships that could be construed as a potential conflict of interest.
